# Electrochemical Properties of Laser-Printed Multilayer Anodes for Lithium-Ion Batteries

**DOI:** 10.3390/nano13172411

**Published:** 2023-08-25

**Authors:** Ulrich Rist, Viktoria Falkowski, Wilhelm Pfleging

**Affiliations:** Institute for Applied Materials-Applied Materials Physics (IAM-AWP), Karlsruhe Institute of Technology (KIT), Hermann-von-Helmholtz-Platz 1, 76344 Eggenstein-Leopoldshafen, Germany

**Keywords:** lithium-ion battery, anode, graphite, silicon, multilayer, laser-induced forward transfer, additive manufacturing

## Abstract

New electrode architectures promise huge potential for improving batteries’ electrochemical properties, such as power density, energy density, and lifetime. In this work, the use of laser-induced forward transfer (LIFT) was employed and evaluated as a tool for the development of advanced electrode architectures. For this purpose, it was first confirmed that the printing process has no effect on the transferred battery material by comparing the electrochemical performance of the printed anodes with state-of-the-art coated ones. For this, polyvinylidene fluoride (PVDF) was used as a binder and n-methyl-2-pyrrolidone (NMP) as a solvent, which is reported to be printable. Subsequently, multilayer electrodes with flake-like and spherical graphite particles were printed to test if a combination of their electrochemical related properties can be realized with measured specific capacities ranging from 321 mAh·g^−1^ to 351 mAh·g^−1^. Further, a multilayer anode design with a silicon-rich intermediate layer was printed and electrochemically characterized. The initial specific capacity was found to be 745 mAh·g^−1^. The presented results show that the LIFT technology offers the possibility to generate alternative electrode designs, promoting research in the optimization of 3D battery systems.

## 1. Introduction

The demand for affordable and improved energy storage systems is increasing due to the EU’s plans to ban the registration of new diesel and petrol cars beginning in 2035 and the expansion of sustainable energies. There are several promising energy storage systems in research, for example, the lithium-ion battery [1], the lithium-sulfur battery [2], the aqueous ammonium-ion battery [3], the Zn-air battery [4], the Zn-ion battery [5], the lithium-selenium battery [6], the sodium-ion battery [7], and supercapacitors [8]. However, the lithium-ion battery is so far the most commonly used one, and introducing new materials and electrode architectures offers the possibility to further improve their electrochemical performance data [9]. The use of silicon as a new anode material is currently in the focus of research as it has an order of magnitude higher theoretical specific capacity (3579 mAh·g^−1^) [10] compared to the state-of-the-art anode material graphite (372 mAh·g^−1^) [11]. However, the enormous volume expansion of silicon during lithiation of up to 300% leads to mechanical degradation of the anode, which subsequently results in a reduced cell lifetime and a reduction in capacity [12]. In comparison, graphite undergoes a volume expansion of 10% during intercalation [13].

In addition to the investigation of new materials, novel electrode architecture concepts promise an approach to increase energy and power density, fast charging capability, as well as cycle stability compared to state-of-the-art batteries [9,11,14]. One of the primary focuses is on the 3D structuring of electrodes [1,15], which can reduce the mechanical stress caused by the volume expansion of silicon during lithiation by creating cavities on a microscale [1,16]. Another approach is the use of multilayered electrodes with respective different materials in each layer to take advantage of the unique properties of each material or layer, such as porosity and particle size [17,18,19].

Besides the generation of new architectures by laser ablation, the printing of electrodes is a promising and versatile method. Here, laser-induced forward transfer (LIFT) offers a reliable tool to print customized electrodes [17,20]. In recent studies, LIFT was used to print anodes for coin cells [17], cathodes, which were subsequently structured [21], and both anodes and cathodes assembled into microbatteries [22].

This work investigated the printing process and its optimization for anodes and compared these printed electrodes to state-of-the-art ones. Therefore, anodes were printed with the LIFT process and were subsequently assembled in coin cells against lithium. After electrochemical formation, the batteries were analyzed with regard to their high-rate capability (“C-rate analysis”). The C-rate is defined as ratio of the applied electrical current—during charging or discharging—and the practical cell capacity, which mainly corresponds to the reciprocal of the time (in hours) required for a complete charging or discharging process at low electrical currents.

**Figure 1:**
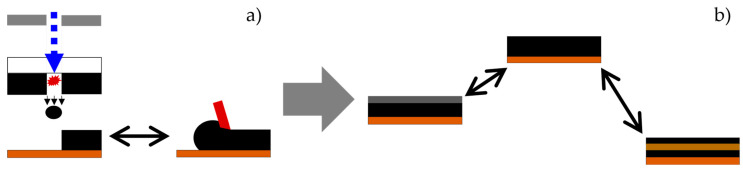
Schematic overview of the presented study. (**a**) First, coated and printed electrodes are compared. (**b**) Afterwards, printed electrodes with pure graphite type are compared with electrodes in a multilayer design containing different active materials.

For the classification regarding state-of-the-art conditions, the printed electrodes were compared with conventionally coated calendered and uncalendered graphite anodes (Figure 1a). The calendered ones were compressed from 50% porosity (uncalendered ones) to 40% porosity to increase the volumetric capacity. As active material, two different types of graphite were used. One is flake-like, and the other is spherical. In addition, the combination of the different material properties in a multilayer electrode was investigated. Finally, a multilayer electrode with a silicon-rich interlayer was printed and subsequently characterized (Figure 1b).

## 2. Materials and Methods

For the anodes, two different types of printing ink were used. One with graphite micro-sized particles as active material and the other with silicon nano-sized particles.

The two employed graphite types for the graphite ink were flake-like graphite (T808, Targray, Kirkland, QC, Canada) and spherical graphite (MCMB: mesocarbon microbeads, PO0120, MSE Supplies, Tucson, AZ, USA). The average particle size of T808 and MCMB is 4.9 µm and 12.2 µm, respectively, measured by laser scattering (LA-950, Horiba, Kyoto, Japan). Conductive carbon black (Super C65) was supplied by TIMCAL, Bodio, Switzerland. Polyvinylidene fluoride (PVDF; Solef 5130, Solvay, Brussels, Belgium) and n-methyl-2-pyrrolidone (NMP, Merck, Darmstadt, Germany) were used to produce the binder solution. For the silicon ink, the silicon nanopowder was supplied by Tagray, Canada (SI-15008, d_50_ = 72 nm).

The graphite printing inks with different kinds of graphite (T808 and MCMB) were prepared according to the reported procedure in [17] with composition taken from [16]. The respective compositions, as well as the composition of the silicon ink, are listed in Table 1.

For the preparation of the graphite ink, the PVDF was dissolved in NMP with a weight ratio of 1:10 in a planetary mixer (SpeedMixer DAC 150 SP, Hauschild, Hamm, Germany) for at least 45 min at rotating speeds from 1000 rpm up to 3500 rpm. Then, the active material and carbon black were added to the planetary mixer and processed at speeds of 1000 rpm up to 3500 rpm for at least 85 min until a homogeneous slurry with the composition of Graphite:PVDF:CB of 85:10:5 was finally obtained.

For the preparation of the silicon ink, the silicon nanoparticles were premixed in a ball mill (Pulverisette 7 premium line, Fritsch, Idar-Oberstein, Germany) with NMP at mixing speeds between 900 rpm and 1050 rpm for 45 min. The PVDF was dissolved in NMP with a concentration of 20 wt % PVDF. Apart from the two exceptions described, the premixes of the binder and the ink were prepared in the same way as the graphite ink.

The viscosity of the prepared slurries was measured with a rheometer (MCR72, Anton Paar Group AG, Graz, Austria) with a plate–plate measuring system. Subsequently, the laser fluence necessary for printing an area corresponding to the specific slurry was determined, as described in more detail in [17]. The values for the viscosity of the inks used and the respective laser fluences are listed in Table 2.

**Figure 2:**
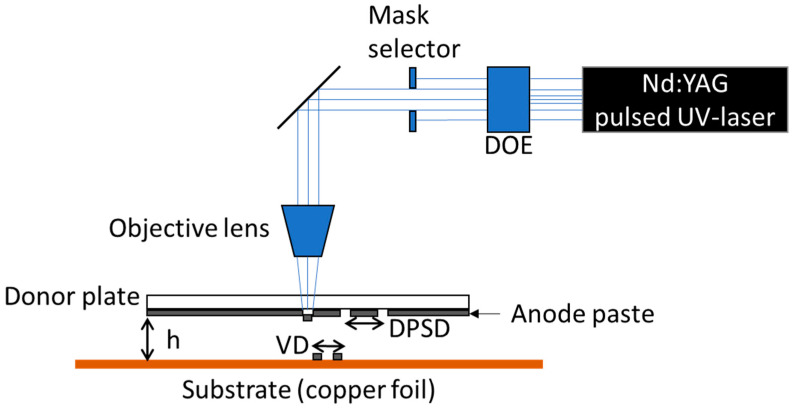
Schematic representation of the setup used for the LIFT process. DOE: diffractive optical element; VD: voxel distance; DPSD: donor plate spot distance; h: high.

The electrodes were printed applying the LIFT (“laser-induced forward transfer”) technique. The process was carried out according to the LIFT process of fluids described by Fernández-Pradas [23]. By imaging the laser on the interface of the donor plate and anode paste, the UV-light absorbing material is partially evaporated, and the expanding gas propels the material in the direction of the substrate. A schematic representation of the setup for the LIFT process and relevant parameters are depicted in Figure 2. For the initial shaping of the laser beam, a diffractive optical element (DOE) is installed in the beam path to transform the Gaussian beam profile into a 2D top-hat intensity profile. Subsequent to the DOE, a mask selector or aperture is introduced to shape the spatial profile of the laser beam into defined circles or rectangles. One parameter is the so-called DPSD (donor plate spot distance), indicating that there is in general no overlap of areas on the donor plate which are exposed to the incident laser beam. A second parameter is the voxel distance (VD) on the substrate, which plays an important role with regard to the established mass (voxel) transfer and realized pattern, e.g., lines or dots. Here, an overlap of around 50% is required for line structure deposition [24]. In addition, the distance h between the anode paste and the substrate surface is essential for the quality of the transferred pattern and the required laser fluence [25].

In this work, a frequency-tripled Nd:YAG laser source (Lumentum, San Jose, CA, USA, Model: Q301-HD-1000R) was applied. It has an operational wavelength of 355 nm, a pulse length of 78 ns, a maximum power of 10 W, and a maximum repetition rate of 30 kHz. A square mask with a resulting laser beam lateral dimension of 83 µm in the imaging plane (interface of donor plate and anode paste) was used. For this purpose, an objective lens demagnifies the selected aperture of the mask selector by a factor of 3.5 onto the backside of the anode paste (compare Figure 2). Prior to the printing process, the respective ink anode paste was deposited on a quartz glass wafer (DSP-200×0675-SGQ-00, Wafer Universe, Elsoff, Germany), which has a diameter of 200 mm and a thickness of 0.675 mm, also referred to as a donor plate. For the anodes, the substrate on which the ink was printed is a 9 µm thick copper foil.

In addition to the printed anodes, state-of-the-art coated graphite anodes were also manufactured from the same slurries as the printed ones for better comparability. Coating was applied with a doctor blade and the thickness was adjusted to have a similar areal capacity loading as the printed ones. Calendered and uncalendered anodes are used for this purpose. The calendered electrodes were compressed to a porosity of 40% and represent the state-of-the-art anodes. Since the printed electrodes are uncalendered, those state-of-the-art anodes that are not calendered are also included in the comparison.

To compare the different types of graphite as well as the printed and coated silicon-containing electrodes, Raman spectroscopy was performed with a Renishaw 1000 system (Renishaw GmbH, Pliezhausen, Germany) equipped with an argon-ion laser (excitation wavelength λ = 514.5 nm, power output P = 23 mW). The output power was attenuated to 25% in order to avoid thermal damage, and data were acquired in the spectral region between 1200 cm^−1^ and 1800 cm^−1^ for the analysis of graphite powder, and a range of 200 cm^−1^ to 2000 cm^−1^ for the analysis of printed and coated silicon electrodes. The latter were manufactured by using the same slurry.

In addition, the distribution of elements in the printed and coated silicon electrodes was analyzed using semiquantitative energy-dispersive X-ray spectroscopy (EDX) on a Phenom XL scanning electron microscope (SEM, PW-100-018, Phenom-World BV, Eindhoven, The Netherlands).

For the electrochemical analyses, electrodes with a diameter of 12 mm were cut out with an ultrashort pulsed (USP) laser operating at a wavelength of 515 µm. Subsequent to the cutting, the electrodes were dried at 100 °C for 24 h in a vacuum oven to evaporate the residual moisture. They were then transferred to an argon-filled glove box (oxygen and water content < 1 ppm) and assembled in coin cells (CR2032) as half-cells versus lithium. The assembled coin cells were electrochemically characterized with a BT 2000 (Arbin Instruments, College Station, TX, USA). In the electrochemical analyses, the cells were cycled symmetrically, so charging and discharging currents are equal. The electrochemical priming of cells with electrodes containing silicon was performed with one C/50 cycle and subsequently three C/20 cycles. The cells with pure graphite electrodes were formed with three C/20 cycles. After electrochemical priming, the batteries were cycled with C-rates ranging from C/10 up to 5C. For the delithiation of the electrodes, meaning charging in the half-cell assembly, the respective battery was charged with a constant current (CC) to the upper cutoff voltage. For the lithiation, i.e., the discharging in half-cells, the battery was first discharged with CC to the lower cutoff voltage and then discharged with a constant voltage (CV) to a defined cutoff current (Table 3). The batteries with graphite anodes were cycled within a voltage window between 0.01 and 1.5 V, whereas the batteries with anodes containing silicon were cycled between 0.06 and 1.5 V to avoid the formation of the crystalline phase (Li_15_Si_4_) and, by this, avoiding a large volume expansion [26]. To establish a certain state of discharge (SOD), the discharge capacity of the last formation cycle was used, see Section 3.1.

The second C/5 cycle at the end of the C-rate measurement was used for comparing the capacity before and after the analysis and calculating the capacity retention (CR) of the battery.

(1) $$CR=\frac{{Capacity}_{C/5,\;\;end}}{{Capacity}_{C/5,\;\;start}}$$

The C-rates, the cutoff current of the CV-phase, and the numbers of cycles are displayed in Table 3. In the presented diagrams, only the first C/5 cycle is shown due to better recognizability.

## 3. Results and Discussion

First, the galvanostatic characterization of cells with printed anodes for both types of graphite are compared to those with coated ones, calendered and uncalendered. Then a multilayer anode with both types of graphite was printed, and the discharge capacity of the assembled cell is presented in comparison to cells with printed anodes using only one type of graphite. At the end, a multilayer anode with a silicon-rich interlayer was printed and compared to the cells assembled with pure and printed graphite anodes.

### 3.1. Printed Graphite Anodes

For this study, electrodes were printed using the LIFT process, which was described in more detail in a previous work [17]. Therefore, printed electrodes were assembled in half-cells versus lithium and galvanostatically characterized with the previously described C-rate analysis (Table 3). To evaluate if the LIFT process modifies the active material in term of its electrochemical properties, the results are compared to results from conventionally coated calendered and uncalendered electrodes. The calendered electrodes were compressed to a porosity of 40% and represent the state of the art.

**Figure 3:**
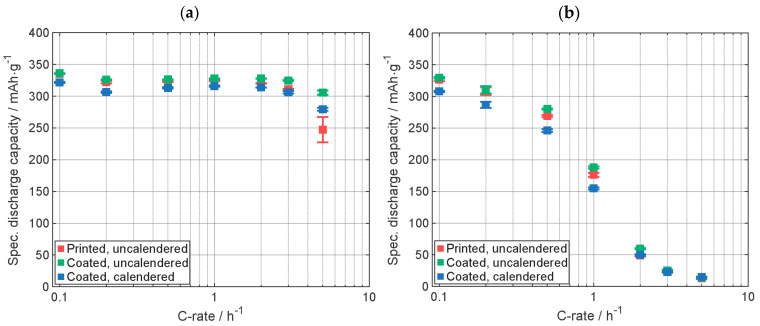
Specific discharge capacity of the C-rate analyses (**a**) with CCCV-phase and (**b**) with only CC-phase. Capacity retention of printed, uncalendered, and calendered electrodes: 101%, 100%, and 102%, respectively (Graphite type: MCMB). All electrodes were manufactured by using the same slurry.

The comparison of the results of C-rate analysis of the cells with MCMB graphite electrodes is presented in Figure 3. Supplementary data of the electrodes are displayed in Table A1. In Figure 3a, the specific discharge capacity with the CCCV-phase is shown. Preliminary results were already reported in [17]. The capacity of the cell with the printed electrode is, except for the highest C-rate, between the capacity of the cells with coated, calendered and the uncalendered ones. The cell with coated, calendered electrode provides, except for the highest C-rate, the lowest discharge capacity, which is due to the lower porosity [27]. In Figure 3b, the specific discharge capacity achieved within the CC-phase is shown. Up to 1C, the specific discharge capacity of the cell with printed electrode is similar to the one with the coated, uncalendered electrode. At 2C, the cell with the printed electrode provided almost the same capacity as the cell with the coated, calendered electrode and a significant smaller discharge capacity compared to the cell with the uncalendered one. Above 2C, all cells have almost the same discharge capacity. The capacity value of the cell with printed electrode is in good agreement with the capacity value of the cells with state-of-the-art coated electrodes. The similar capacity of the cells with the state-of-the-art coated electrodes and the cell with the printed electrode suggests that the active material was not modified during the LIFT process at the applied laser fluence.

**Figure 4:**
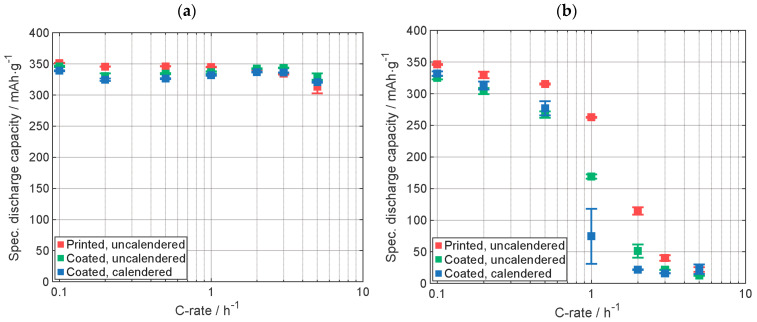
Specific discharge capacity of the C-rate analyses (**a**) with CCCV-phase and (**b**) with only CC-phase. Capacity retention of printed, uncalendered, and calendered electrodes: 99%, 104%, and 103%, respectively (Graphite type: T808). All electrodes were manufactured by using the same slurry.

Figure 4 shows the comparison of the results of the C-rate analysis of the cells with T808 graphite electrodes. Supplementary data on the electrodes are listed in Table A2. In Figure 4a, the results for the specific discharge capacity are shown for the whole discharge cycle, including the CV-phase. The cell with the printed electrode displays the highest specific discharge capacity for C-rates up to 1C. For C-rates larger than 1C, all cells show very similar discharge capacities. At lower C-rates, the cells with calendered electrodes provide the lowest discharge capacity, similar to the results for the electrodes with the MCMB graphite shown in Figure 3. In Figure 4b, only the specific discharge capacity of the CC-phase is shown. For each C-rate, except for 5C where all cells have similar capacities, the cell with the printed electrode outperforms the other electrodes in terms of discharge capacity. One explanation of the high specific capacity of the cell with the printed electrode could be that during the LIFT process, solid material like the binder was also partially evaporated, the cause of the higher fluence needed to be compared to the ink with MCMB, and so the relative content of the solids might change. However, due to the high specific discharge capacity, it can be suggested that the active material was not modified. The lower discharge capacity of the calendered electrode is most likely due to the lower porosity of the electrode [27] (Table A2).

**Figure 5:**
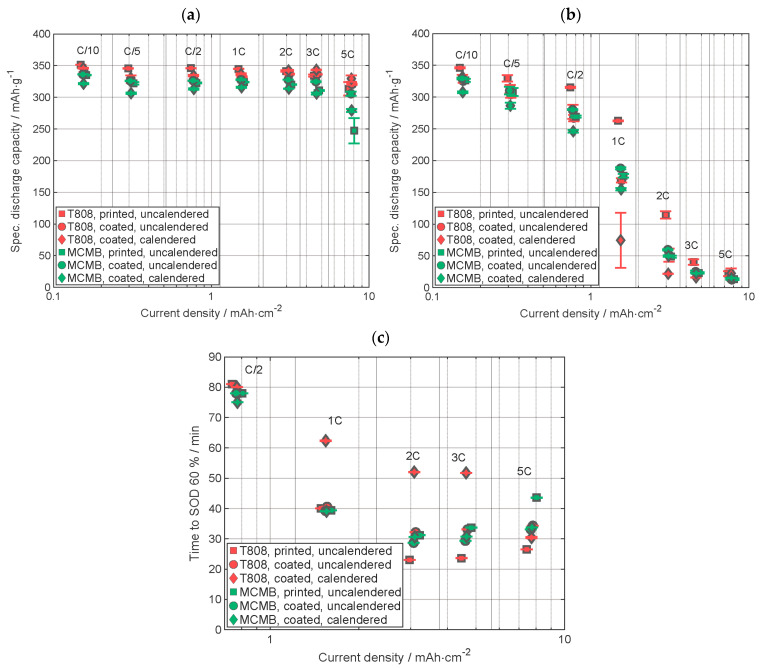
Comparison of electrochemical data of graphite-containing cells, (**a**) the specific discharge capacity including the CCCV-phase, (**b**) the specific discharge capacity only including the CC-phase, and (**c**) the duration required to discharge 60% of the discharge capacity reached in the last cycle of the formation of the electrodes.

**Figure A1:**
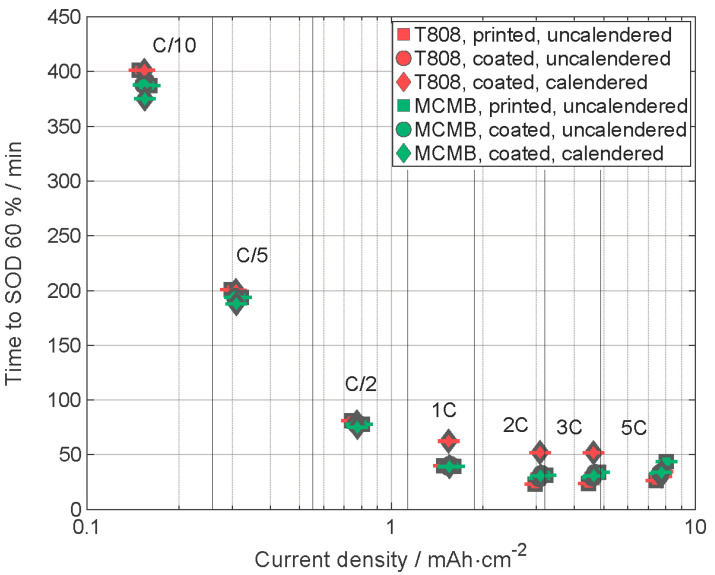
Time to SOD 60% of the last discharge cycle of the formation. C/10 and C/5 are added compared to Figure 5c.

The comparative results of the electrochemical analyses for the electrodes with the two graphite types are presented in Figure 5. The specific discharge capacity from CCCV data (Figure 5a) shows that the cells with T808 graphite electrodes have a slightly higher discharge capacity than those with the MCMB graphite electrodes at each C-rate. This can be attributed to the smaller particle size of the T808 graphite [28] and slight differences at the structural level of the graphite, which will be discussed later. Considering the capacity measured during a CC discharge (Figure 5b), the cell with printed uncalendered T808 graphite electrodes shows the highest discharge capacity up to a C-rate of C/2, and the cell with coated calendered MCMB electrodes shows the lowest discharge capacity. The specific discharge capacities of the other batteries are very similar to each other. At 1C, there is a huge difference in specific capacity between the cell with printed and those with coated, calendered T808 graphite electrodes, while the capacity of the cell with coated, uncalendered electrodes provides capacity values intermediate between these two. The difference of the specific discharge capacities of the cells with MCMB electrodes are small compared to the differences of those with electrodes containing T808. At higher C-rates, the differences are getting smaller, and with 5C, the capacities are very similar to each other. Even though the discharge capacity of cells containing MCMB with CV-phase is smaller than those with T808, a comparable capacity is achieved in the CC-phase. The higher the capacity in the CC-phase, the shorter the discharge time, since a higher electrical current is applied to the cells in the CC-phase than in the CV phase. Figure 5c displays the discharging time up to a state of discharge of 60%, calculated as described above. In Figure A1, in the Appendix A, the data from C/10 are shown. For the C-rates from 1C to 3C, the cell with coated, calendered T808 electrode needs almost twice the time to reach SOD 60% compared to the other cells. The cell with printed T808 electrode needs, from 2C, the shortest time to reach SOD 60%. The respective time differences for cells with MCMB electrodes are very similar to each other except for 5C, where about 10 min more are needed to discharge the cell with the printed electrode to a SOD of 60% compared to the other ones. The time needed to discharge 60% of the cell with the coated, uncalendered T808 electrode is all the time very similar compared to the ones of the electrodes containing MCMB.

**Figure 6:**
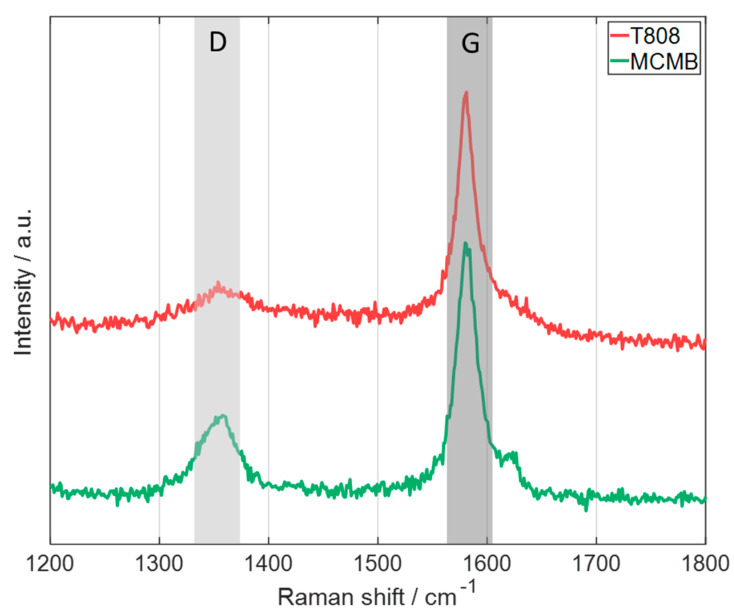
Raman spectra of the pristine graphite powders of the types of graphite used showing the D and G bands (laser excitation wavelength: 514.5 nm).

To further compare the T808 and the MCMB graphite, Raman spectroscopy was performed on the pristine graphite powders (Figure 6). The position of the peak of the G band of both types of graphite is quite similar and also in the graphite-related G band range [29]. For the D band, which is referred to as the disorder band, the peak of the MCMB is well pronounced compared to the peak of the T808 graphite, which is almost negligible compared to the noise of the measurement (graphite-related D band at about 1345 cm^−1^ [29]). The integrated intensity ratio of the D band to the G band is assigned to the defect quantity in graphitic materials [29,30,31], suggesting that the T808 flake-like graphite has fewer defects in its graphite structure than the MCMB graphite, which could be also an explanation of the slightly higher specific capacity of the T808-based anode (Figure 5a). It can be concluded that both types of active materials, T808 and MCMB, are mainly providing graphitic modification with slight differences at the structural level and in terms of the basal plane orientation.

### 3.2. Multilayer Graphite Anode

Multilayer electrodes incorporating T808 and MCMB layers were printed and analyzed to determine the feasibility of combining the advantageous properties of the different graphite types.

**Figure 7:**
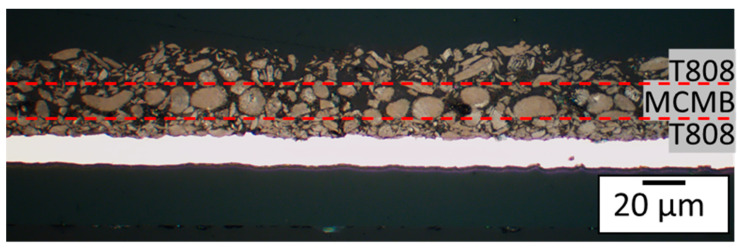
Light microscope cross-sectional image of an embedded multilayer electrode with two different graphite types. Order (from top to bottom): one layer of T808, one layer of MCMB, and one layer of T808 (Different order than for the electrode for the electrochemical characterization of the multilayer).

Figure 7 displays a cross-sectional view of a printed multilayer electrode with one layer of MCMB between two layers of T808. This printing sequence was used to provide a clear distinction of each layer. In the electrode for the electrochemical characterization, the printing order was two layers of T808 and one layer of MCMB at the top, in the following called ML1. At higher C-rates, the upper materials are more important to the electrochemical performance of the battery. This is due to the fact that the lower graphite particles are not lithiated as the penetration depth of the lithium becomes smaller at higher electrical currents [32]. Based on this, the configuration was chosen because MCMB showed a good discharge capacity in the CC-phase (see Figure 5), and the electrodes with T808 showed a higher capacity when including a CV-phase after the CC-phase.

**Figure 8:**
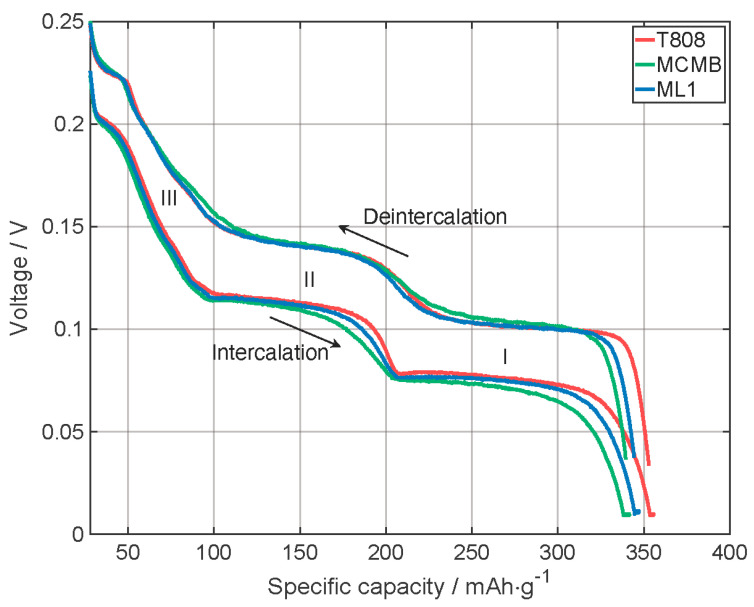
Comparison of the cell performance of electrodes with T808, MCMB, and the multilayer electrode ML1 with two layers T808 and one MCMB layer on top, at the last formation cycle: voltage profiles as a function of the specific capacity for CCCV-discharging and CC-charging. (Stage III: C_24_Li → C_18_Li; Stage II: C_18_Li → C_12_Li; Stage I: C_12_Li → C_6_Li, see [33]).

**Figure A2:**
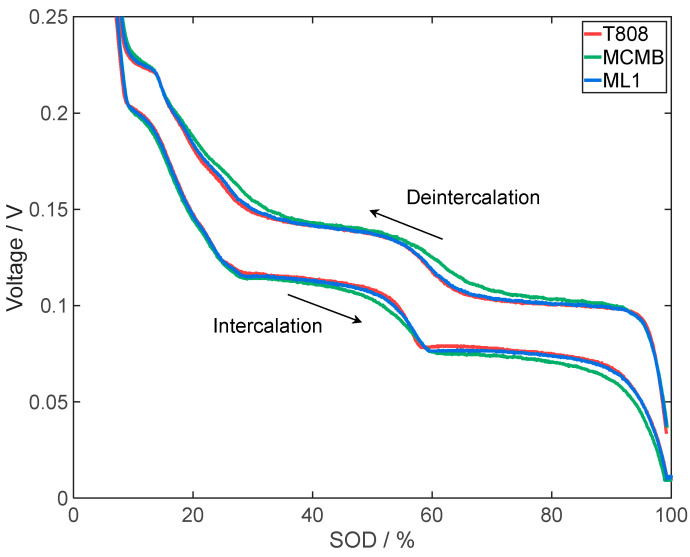
Comparison of the cell performance of electrodes with T808, MCMB, and the multilayer electrode ML1 with two layers T808 and one layer MCMB on top, at the last formation cycle: voltage profiles as a function of the SOD for CCCV-discharging and CC-charging.

**Figure A3:**
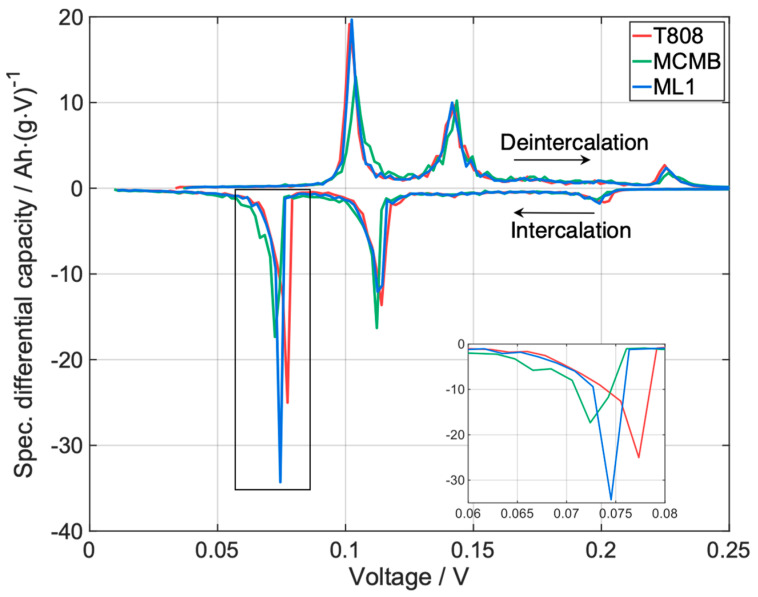
Comparison of the cell performance of electrodes with T808, MCMB, and the multilayer electrode ML1 with two layers T808 and one layer MCMB on top, at the last formation cycle: specific differential capacities dq/dV as a function of the voltage of the charging and discharging, an inset showing a magnification of the last peak of the intercalation.

Figure 8 provides a closer look at the last cycle of the formation (3rd cycle) of the cells with the printed MCMB, T808, and ML1 electrode. For all three electrode architectures, all three intercalation and deintercalation plateaus related to graphite are visible, as reported in [33,34,35]. The voltage at which the plateaus are visible can be detected better in Figure A3, where the specific differential capacity is displayed as a function of the voltage. In addition, in Table A3, the voltage values of the peaks are listed. For the intercalation steps, the voltage of the ML1 electrode is always between the values of T808 and MCMB, whereby the plateaus of MCMB are at a slightly lower voltage than the T808 ones, and thus, a slightly higher overpotential when reaching the intercalation voltage is needed. In addition, the plateaus of MCMB are less pronounced than the plateaus of T808, which could be explained by the differences on the structural level as described above, but geometry and the particle size could also have an influence. During delithiation, the multilayer electrode ML1 follows more closely the progress of the T808 graphite electrode. The delithiation process of MCMB generally occurs at slightly higher voltage plateaus than the other two electrodes at the same specific capacity, and as with lithiation, the plateaus of cells with MCMB are also less pronounced in delithiation, which could be explained as above. Neglecting the slightly higher capacitance of the cells with the T808 and the ML1 electrodes compared to the cell with the MCMB electrode and comparing the voltage profile as a function of SOD, the values of the cell with the ML1 electrodes fit more closely to the values of the cell with the T808 electrode (Figure A2). Since the T808 makes up about two-thirds of the electrode layer, it can be concluded that it was possible to merge the electrochemical properties with this multilayer design.

**Figure 9:**
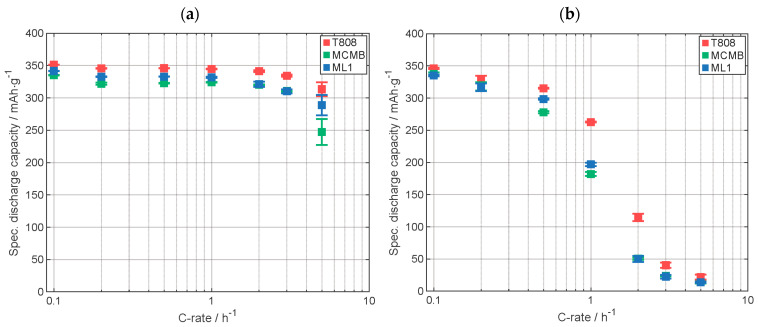
Specific discharge capacity of the C-rate analyses (**a**) with CCCV-phase and (**b**) with only CC-phase. Capacity retention of T808, MCMB, and ML1 electrodes: 99%, 101%, and 100%, respectively (all electrodes were printed).

Rate capability tests of cells with printed electrodes made of T808, MCMB, and ML1 were performed to compare the capacity retention of the different types (Figure 9). Specifications of the used electrodes are displayed in Table A4. For C-rates up to 1C, the discharge capacity of the cell with ML1 electrode measured including the CV-phase, as seen in Figure 9a, is lying between the capacity of the electrode with T808 and MCMB, with MCMB having the lower specific discharge capacity. At 2C and 3C, the capacity of the cell with multilayer electrode ML1 is similar to the capacity of the cell with the MCMB electrode. At 5C, the value of the capacity of the ML1 electrode cell is between the capacity values of the other two cells. Here, however, the standard deviations are too large for a meaningful comparison. In Figure 9b, the specific discharge capacity of the CC-phase is displayed. For the C-rates C/10 and C/5, the discharge capacity of the ML1 cell is similar to the one of the cell with MCMB electrode. From C/2 to 1C, the capacity of the cell with ML1 electrode is between the capacity of the cells with the other electrodes. From 2C up to 5C, the capacity of the cell with ML1 electrode is similar to the specific discharge capacity of the cell with MCMB electrode. So, it could be shown, that at higher currents, the electrochemical performance of the upper layer plays a more important role due to the fact that with increasing C-rate the lithium penetration depth along the electrode cross section, from the top of the electrode in the direction to the current collector, is decreasing [32].

### 3.3. Multilayer Silicon Anode

**Figure 10:**
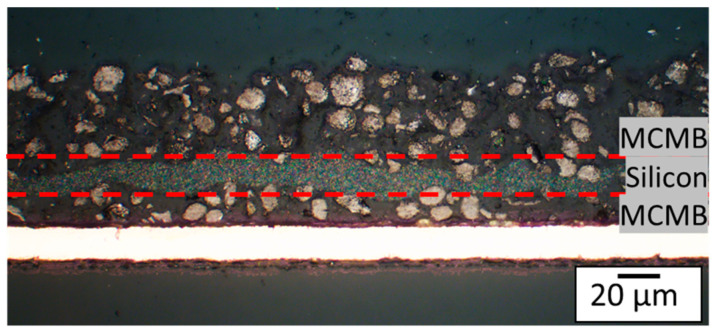
Light microscope cross-sectional image of an embedded multilayer electrode with a silicon-enriched layer sandwiched between two layers of graphite. Order from bottom to top: one layer of MCMB, two layers of Si, and one layer of MCMB. This electrode will further be referred to as ML2.

In research, the most common way to add silicon to an electrode is by mixing it into the graphite slurry, producing a blended electrode [9,10,16,36,37,38]. Apart from this classical approach, silicon is now implemented in the electrode as a silicon-enriched layer in between two graphite layers, as shown in Figure 10. This electrode design could help to maintain the integrity of the graphite matrix during volume expansion of the silicon during lithiation, thereby preventing any matrix cracking.

Figure 10 shows a cross section of a multilayer electrode, referred to as ML2 in the following, with an intermediate silicon-rich layer with an average layer thickness of 11 µm and an overall silicon content in the anode of 12 wt %. To determine the weight and subsequently calculate the specific capacity of ML2, the weight of the silicon in the electrode was derived by printing three silicon reference samples with the same conditions as used for ML2, weighing them, and calculating their average value. This silicon contribution to the weight was then subtracted from the total weight of ML2, allowing to identify the contribution of graphite. Using this approach, the specific discharge capacity for the last formation step was found to be 745 mAh·g^−1^. However, the capacity continues decreasing with each subsequent cycle.

**Figure 11:**
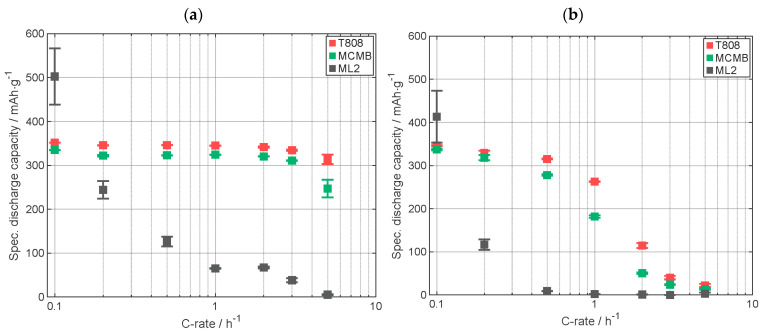
Specific discharge capacity of the C-rate analyses: (**a**) with CCCV-phase, (**b**) with CC-phase. Capacity retention of T808, MCMB, and ML2 electrodes: 99%, 101%, and 84%, respectively (all electrodes were printed).

In order to assess how well the silicon-containing printed multilayer electrode ML2 performed compared to electrodes based on MCMB and T808, C-rate analyses were conducted at different C-rates displayed in CCCV (Figure 11a) and CC (Figure 11b) mode. In both diagrams in Figure 11, at a C-rate of C/10, the capacity of the cell with ML2 is higher than those with graphite electrodes, but a large decrease in capacity of ML2 in the following C-rate (C/5) occurs, while dropping below the capacity values of the cells with graphite electrodes. Figure 11b shows the specific discharge capacity of the CC-phase, and it can be observed that at a C-rate of C/2 and above, almost no capacity is provided during discharge. As described earlier, a smaller capacity in the CC-phase compared to the CCCV-phase indicates slower intercalation. The large capacity fading of ML2 cell at low C-rates, below C/5, and the low capacity retention of 84% display a degradation of the electrode material. It is worth noticing that the capacity retention is calculated with the C/5 cycles, and that at the first C/5 cycle sequence, a tremendous degradation has already occurred. This shows that the intermediate Si layer increased the initial capacity of the electrode but during electrochemical cycling capacity fading occurs. The degradation could be explained by the impact of the used binder. In other studies, it has been shown that PVDF is not a suitable binder for silicon-containing electrodes due to weak interactions between binder and silicon particles, leading to a contact loss of the particles in the electrode matrix or a capacity fading caused by a strongly grown solid electrolyte interface [39,40,41,42,43]. More specifications on the batteries are displayed in Table A5.

To better understand whether the rapid degradation of the silicon was caused by the unsuitable PVDF binder or whether the silicon was damaged or modified by the laser irradiation during the LIFT process, further analyses were carried out by comparing printed silicon electrodes with state-of-the-art coated ones before cycling directly after preparation.

**Figure 12:**
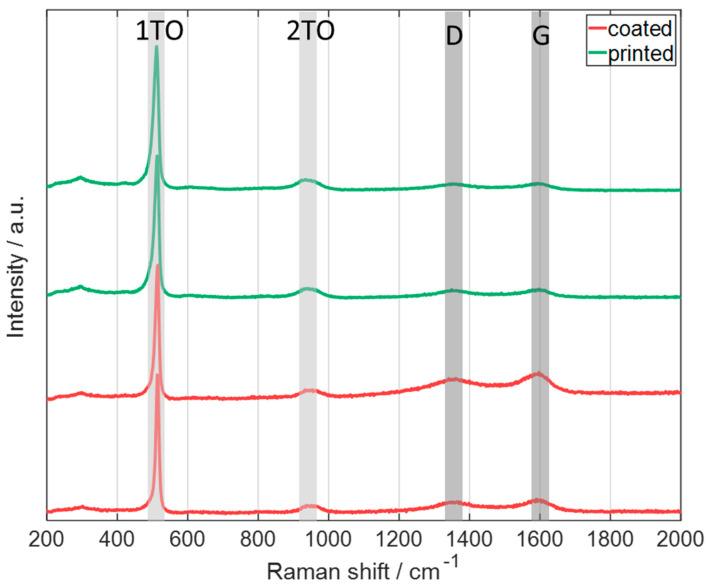
Raman spectra of pristine silicon electrodes (red line: coated electrodes; green line: printed electrodes), showing the D and G bands of graphite and the 1TO and 2TO of crystalline silicon (laser excitation wavelength: 514.5 nm). All electrodes were manufactured by using the same slurry.

First, Raman spectra of state-of-the-art coated silicon electrodes and printed silicon ones were obtained, as displayed in Figure 12. For each electrode, two measurements at different positions are shown. In the Raman spectra, the 1TO and 2TO bands can be observed, which are associated with crystalline silicon as described in [44]. Additional to the silicon peaks, also the graphite-related bands, D and G, are displayed in the spectrum. These peaks originate from the carbon black, the conductive additive in the electrode. Comparing the spectra, no additional bands or significant shifts can be observed, leading to the assumption that no modification of the silicon particles occurs during the LIFT process.

**Figure A4:**
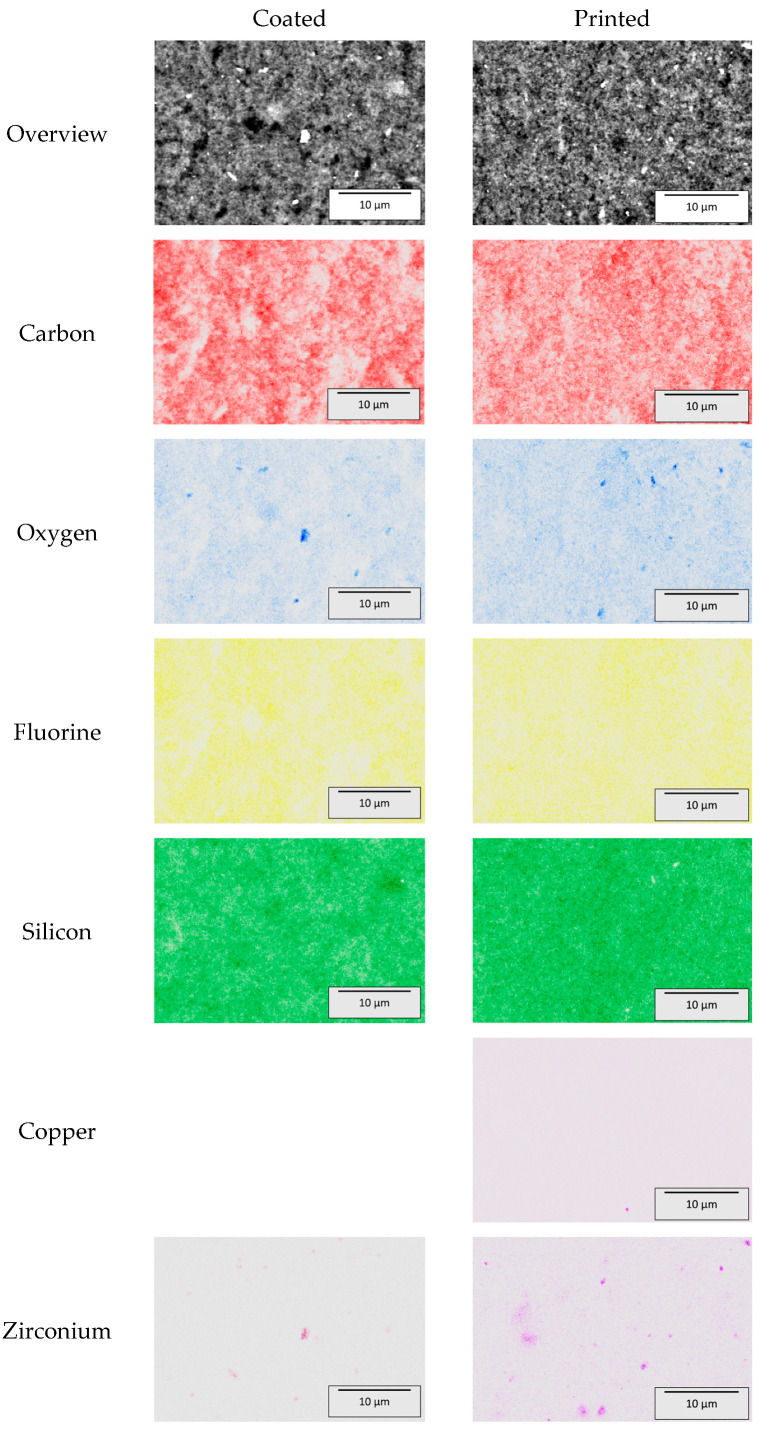
SEM images (“overview”) of pristine, coated and printed, silicon electrodes, and respective elemental mapping achieved by EDX. Elemental mapping to the results displayed in Table 4.

In addition to Raman spectroscopy, EDX elemental mapping measurements were performed to determine the distribution of elements in the differently manufactured electrodes. The results of the measurements are presented in Table 4, and the corresponding element distribution maps are presented in Figure A4.

The copper, which is detected in the printed electrode, is due to an incomplete coverage of the copper current collector by the electrode material. The detected zirconium oxide particles in the electrodes are impurities originating from the ball milling of the silicon, in which the lining of the containers and the milling balls are made of zirconia. The fluorine measured in the electrodes comes from the PVDF binder and the carbon from the carbon black and binder. As for the oxygen, it arises from both the zirconia and the surface of the silicon nanoparticles. On the surface of silicon particles, a native oxide layer is developed when exposed to ambient air [16]. The measured silicon contents of 41.7 wt % and 43.1 wt % closely align with the 40 wt % of silicon added to the slurry. As the sensitivity of an EDX measurement for light elements is low, the small differences in oxygen, carbon, and fluorine values between the printed and coated electrodes are negligible. It can be concluded that no significant distinction between the printed and the coated electrodes can be observed.

## 4. Conclusions

The LIFT process was realized to print graphite anodes which were subsequently assembled in coin cells. To evaluate if the material is modified with regard to its electrochemical properties during LIFT, a comparative study with classical state-of-the-art coated anodes was performed. Due to the fact that the printed anodes are uncalendered, classical anodes without calendering were also produced in addition to the state-of-the-art calendered ones. For this purpose, a flake-like and a spherical graphite type were used. For both types of graphite, it was shown that a comparable specific discharge capacity could be achieved with the printed electrodes, which leads to the conclusion that no modification of the active material during the LIFT process occurs. Further, a multilayer graphite anode was prepared to combine the properties of the two used graphite types. For this purpose, two layers of T808 with one layer of MCMB on top were printed. The electrochemical performance during the C-rate analysis of the cell with this multilayer electrode, referred to as ML1, was compared with the ones of the printed anodes with only one type of graphite. It was shown that at low electrical currents (C/10), the specific discharge capacity of the cell with multilayer electrode is laying between the values of the two anodes. At high currents, the capacity of the cell with multilayer electrode approaches the capacity of the top layer, namely MCMB, because the entire electrode is no longer fully utilized due to a smaller lithium penetration depth [32]. In addition to the graphite multilayer electrode, a multilayer electrode with an intermediate silicon-rich layer was printed, referred to as ML2. Raman spectroscopy and EDX measurements showed no significant difference between the two differently prepared (“printed” and “coated”) electrode types, which were prepared with silicon-rich ink only. For ML2, a specific discharge capacity of 745 mAh·g^−1^ was achieved at the end of the electrochemical priming, but a tremendeous capacity fading during cycling was observed, which is due to a fast mechancial degradation of the electrode. This is mainly assigned to the used PVDF binder, which was reported not to be suitable for electrodes containing silicon because of the weak interactions with the silicon particles [39,40,41,42,43]. Therefore, in ongoing studies it is essential to switch to alternative binders such as polyacrylic acid, sodium carboxymethylcellulose, and styrene-butadiene rubber, which would be more suitable [40,41,43]. Development work is still required making slurries with these binders printable, as the water used as a solvent with the aforementioned binders evaporates rapidly.

## Figures and Tables

**Table 1:** Composition of the graphite and silicon inks.

Material Graphite Ink	Mass Fraction /wt %	Material Silicon Ink	Mass Fraction /wt %
Graphite	85	Silicon	40
PVDF	10	PVDF	20
Carbon black	5	Carbon black	40

**Table 2:** Measured ink viscosities and laser fluences for voxel transfer.

Ink	Viscosity (@ 50 s^−1^) /Pa·s	Laser Fluence /J·cm^−2^
T808 graphite	23.41	1.30
MCMB graphite	16.47	1.06
Silicon	3.30	0.52

**Table 3:** C-rates, cutoff current of the electrochemical priming (formation) and the C-rate analysis, and number of cycles. C/50 cycle is only applied if the electrode contains silicon.

**C-rate**	C/50	C/20	C/10	C/5	C/2	1C	2C	3C	5C	C/5
**Cutoff current (CV)**	C/100	C/50	C/20	C/10	C/10	C/10	C/10	C/10	C/10	C/10
**Number of cycles**	1	3	5	5	10	10	10	10	10	5

**Table 4:** Results from the EDX measurements of the two pristine, printed and coated, silicon electrode materials. For better comparability, the results from the printed electrode are additionally calculated excluding copper. All electrodes were manufactured by using the same slurry.

Element	Coated		Printed		Printed Excl. Copper	
	Atomic %	Weight %	Atomic %	Weight %	Atomic %	Weight %
C	71.877	53.5	68.929	48.6	69.671	51.3
O	2.723	2.7	3.620	3.4	3.713	3.6
F	1.359	1.6	1.255	1.4	1.287	1.5
Si	23.953	41.7	24.620	40.6	25.252	43.1
Cu	-	-	1.501	5.6	-	-
Zr	0.088	0.5	0.075	0.4	0.077	0.4

## Data Availability

Data sharing is not applicable to this article.

## Appendix A

**Table A1:** Specifications of the electrodes in Figure 3, graphite: MCMB.

Electrode	Measured Areal Capacity /mAh·cm^−2^	Porosity /%	Capacity Retention /%	Initial Coulombic Efficiency /%	Electrode Thickness /µm	Ohmic Resistance ^1^ /Ω·cm^2^
Printed, uncalendered	1.68	49	101	91.6	50.0	99.5
Coated, uncalendered	1.60	48	100	91.7	47.9	82.0
Coated, calendered	1.56	40	102	91.2	40.9	109.7

^1^ Ohmic resistance was measured with a current of 0.1 mA and a pulse time of 1 ms.

**Table A2:** Specifications of the electrodes in Figure 4, graphite: T808.

Electrode	Measured Areal Capacity /mAh·cm^−2^	Porosity /%	Capacity Retention /%	Initial Coulombic Efficiency /%	Electrode Thickness /µm	Ohmic Resistance ^1^ /Ω·cm^2^
Printed, uncalendered	1.60	52	99	90.6	48.8	70.0
Coated, uncalendered	1.64	52	103	91.0	51.2	105.0
Coated, calendered	1.66	40	104	89.9	40.6	96.8

^1^ Ohmic resistance was measured with a current of 0.1 mA and a pulse time of 1 ms.

The specific differential capacities were calculated by taking the capacity data from the respective galvanostatic measurements, which are saved with certain time intervals of one minute. Then, they were filtered by certain voltage steps, here 2 mV, due to the fact that the noise in the raw data will become an issue by the differentiation step [45].

**Table A3:** Voltage for the peaks of the differential capacity shown in Figure A3, from low voltage to high voltage.

Electrode	Deintercalation			Intercalation		
	Peak I /V	Peak II /V	Peak III /V	Peak I /V	Peak II /V	Peak III /V
T808	0.102	0.143	0.225	0.077	0.114	0.201
MCMB	0.104	0.144	0.226	0.072	0.112	0.198
ML1	0.102	0.142	0.225	0.075	0.113	0.199

**Table A4:** Specifications of the electrodes assigned to Figure 9.

Electrode	Measured Areal Capacity /mAh·cm^−2^	Porosity /%	Capacity Retention /%	Initial Coulombic Efficiency /%	Electrode Thickness /µm	Ohmic Resistance ^1^ /Ω·cm^2^
T808	1.60	52	99	90.6	48.8	70.0
MCMB	1.68	49	101	91.6	50.0	99.5
ML1	1.78	53	100	90.4	57.8	78.4

^1^ Ohmic resistance was measured with a current of 0.1 mA and a pulse time of 1 ms.

**Table A5:** Specifications of the electrodes assigned to Figure 11.

Electrode	Measured Areal Capacity /mAh·cm^−2^	Silicon Content /%	Capacity Retention /%	Initial Coulombic Efficiency /%	Electrode Thickness /µm	Ohmic Resistance ^1^ /Ω·cm^2^
T808	1.60	0	99	90.6	48.8	70.0
MCMB	1.68	0	101	91.6	50.0	99.5
ML2	2.82	12	84	78	66.5	76.5

^1^ Ohmic resistance was measured with a current of 0.1 mA and a pulse time of 1 ms.
